# Marriage Laws

**Published:** 1898-09

**Authors:** 


### Marriage Laws.

Last week Representative Parker introduced a bill in the Ohio Legislature which has for its purpose a regulation of marriage which is of very great import to the people of not only this State, but its enactment into a law and reasonable enforcement would surely be followed by similar enactments in other States.

The bill provides that all persons seeking marriage license shall be examined by a board of three physicians, to be appointed by each county Probate Judge, who shall examine such persons to see that they are free from insanity, dipsomania, tuberculosis, cancer, venereal and other hereditary diseases, and are not criminals.

The bill is a reasonable and just one, and represents a sentiment already expressed in the pages of this journal. That such a law would be productive of great good, reduce suffering and mortality rates there can be no question. Modern medical science has attained such a degree of perfection as to warrant such measures.—*The Cincinnati Lancet-Clinic.*

